# IL‐34 deficiency impairs FOXP3^+^ Treg function in a model of autoimmune colitis and decreases immune tolerance homeostasis

**DOI:** 10.1002/ctm2.988

**Published:** 2022-08-28

**Authors:** Antoine Freuchet, Apolline Salama, Séverine Bézie, Laurent Tesson, Séverine Rémy, Romain Humeau, Hadrien Règue, Céline Sérazin, Léa Flippe, Pärt Peterson, Nadège Vimond, Claire Usal, Séverine Ménoret, Jean‐Marie Heslan, Franck Duteille, Frédéric Blanchard, Magali Giral, Marco Colonna, Ignacio Anegon, Carole Guillonneau

**Affiliations:** ^1^ Nantes Université, CHU Nantes, CNRS, INSERM, Center for Research in Transplantation and Translational Immunology UMR 1064, ITUN5 Nantes F‐44000 France; ^2^ Institute of Biomedicine and Translational Medicine University of Tartu Tartu Estonia; ^3^ CHU Nantes, Inserm, CNRS, SFR Santé, Inserm UMS 016, CNRS UMS 3556 Nantes Université Nantes France; ^4^ Chirurgie Plastique Reconstructrice et Esthétique CHU Nantes Nantes France; ^5^ INSERM UMR1238 Bone Sarcoma and remodeling of calcified tissues Nantes University Nantes France; ^6^ Department of Pathology and Immunology Washington University School of Medicine St. Louis Missouri USA

**Keywords:** autoimmunity, CRISPR/Cas9, Foxp3, IL‐34, immunotherapy, knockout, rat, tolerance, Treg

## Abstract

**Background:**

Immune homeostasis requires fully functional Tregs with a stable phenotype to control autoimmunity. Although IL‐34 is a cytokine first described as mainly involved in monocyte cell survival and differentiation, we recently described its expression by CD8^+^ Tregs in a rat model of transplantation tolerance and by activated FOXP3^+^ CD4^+^ and CD8^+^ Tregs in human healthy individuals. However, its role in autoimmunity and potential in human diseases remains to be determined.

**Methods:**

We generated *Il34* ^−/−^ rats and using both *Il34* ^−/−^ rats and mice, we investigated their phenotype under inflammatory conditions. Using *Il34* ^−/−^ rats, we further analyzed the impact of the absence of expression of IL‐34 for CD4^+^ Tregs suppressive function. We investigated the potential of IL‐34 in human disease to prevent xenogeneic GVHD and human skin allograft rejection in immune humanized immunodeficient NSG mice. Finally, taking advantage of a biocollection, we investigated the correlation between presence of IL‐34 in the serum and kidney transplant rejection.

**Results:**

Here we report that the absence of expression of IL‐34 in *Il34* ^−/−^ rats and mice leads to an unstable immune phenotype, with production of multiple auto‐antibodies, exacerbated under inflammatory conditions with increased susceptibility to DSS‐ and TNBS‐colitis in *Il34* ^−/−^ animals. Moreover, we revealed the striking inability of *Il34* ^−/−^ CD4^+^ Tregs to protect Il2rg^−/−^ rats from a wasting disease induced by transfer of pathogenic cells, in contrast to *Il34* ^+/+^ CD4^+^ Tregs. We also showed that IL‐34 treatment delayed EAE in mice as well as GVHD and human skin allograft rejection in immune humanized immunodeficient NSG mice. Finally, we show that presence of IL‐34 in the serum is associated with a longer rejection‐free period in kidney transplanted patients.

**Conclusion:**

Altogether, our data emphasize on the crucial necessity of IL‐34 for immune homeostasis and for CD4^+^ Tregs suppressive function. Our data also shows the therapeutic potential of IL‐34 in human transplantation and auto‐immunity.

**Highlights:**

– Absence of expression of IL‐34 in *Il34^−/−^* rats and mice leads to an unstable immune phenotype, with a production of multiple auto‐antibodies and exacerbated immune pathology under inflammatory conditions.
– *Il34^−/−^* CD4^+^ Tregs are unable to protect *Il2rg^−/−^* rats from colitis induced by transfer of pathogenic cells.
– IL‐34 treatment delayed EAE in mice, as well as acute GVHD and human skin allograft rejection in immune‐humanized immunodeficient NSG mice.

## Graphical abstract

The impact of an IL‐34 deficiency at steady state, upon challenges and its use as a therapeutic tool.

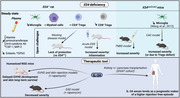

## 1 INTRODUCTION

IL‐34 is a homodimeric cytokine binding three distinct receptors or co‐receptors, namely CSF‐1R (CD115), CD138 (syndecan‐1) and PTPζ.^1^, ^2^ CSF‐1 is another ligand of CD115 but binds with a lower affinity than IL‐34, and they both induce survival and differentiation of monocytes towards type 2 ‘regulatory’ macrophages.^3^, ^4^ However, IL‐34 and CSF‐1 spatio‐temporal expressions are mainly distinct, and CSF‐1 does not bind to CD138 and PTPζ.^5^ Thus, they have been shown to exert also non‐overlapping roles.^5^

We have recently demonstrated that IL‐34, but not CSF‐1, is significantly overexpressed in antigen‐specific CD8^+^ Tregs from long‐term tolerant transplanted rats (treated with CD40Ig, a chimeric molecule blocking the CD40‐CD40L pathway) versus CD8^+^ Tregs from naive rats.^6^, ^7^, ^8^ Further analysis showed that IL‐34 expression in human T cells is restricted to, not only activated FOXP3^+^ CD8^+^ Tregs, but also to FOXP3^+^ CD4^+^ Tregs with about 50% of the FOXP3^+^ Tregs expressing IL‐34.^6^, ^9^ We also showed that treatment using an AAV encoding IL‐34 alone or together with a short suboptimal dose of rapamycin efficiently delayed or inhibited allograft rejection in vivo in rats through differentiation of tolerogenic macrophages responsible, in turn, for induction of CD4^+^ and CD8^+^ Tregs. In this model, the CD4^+^ and CD8^+^ Tregs were responsible for the long‐term tolerance induction effect of the treatment. In vitro, we showed the IL‐34 inhibited both rats and humans effector CD4^+^CD25^−^ T cells proliferation and induced human Foxp3^+^ CD4^+^ and CD8^+^ Tregs expansion.^6^, ^10^ In addition, a suppressive role for IL‐34 was also demonstrated by others^11^ in rodent liver transplantation models.

Although *Il34^LacZ/LacZ^* mice have been studied and no spontaneous autoimmunity or inflammatory diseases were reported,^12^ we speculated that it could be due to lack of an inflammatory/autoimmune stimulus. Indeed, in human, increased IL‐34 serum levels have been associated with inflammatory diseases^1^, ^2^; however, IL‐34 role was not properly described and mostly circumstantial. Thus, the contribution of IL‐34 in immune homeostasis, Treg function, as well as its role in autoimmunity and human transplant rejection remains unclear and needs to be further addressed. Herein, we generated *Il34^−/−^* rats and using both *Il34^−/−^* rats and mice, we investigated their phenotype under inflammatory conditions. Using *Il34^−/−^* rats, we further analysed the impact of the absence of expression of IL‐34 for CD4^+^ Tregs suppressive function. We investigated the potential of IL‐34 in human disease to prevent xenogeneic GVHD and human skin allograft rejection in immune humanized immunodeficient NSG mice. Finally, taking advantage of a biocollection, we investigated the correlation between presence of IL‐34 in the serum and kidney transplant rejection.

Altogether, our study emphasizes the crucial role of IL‐34 for immune homeostasis and for CD4^+^ Tregs suppressive function and the therapeutic potential of IL‐34 in human transplantation and autoimmunity.

## 2 RESULTS

### 2.1 At homeostasis, IL‐34 participates in microglia development and regulates cytokines, enzymes and auto‐antibodies production

**FIGURE 1:**
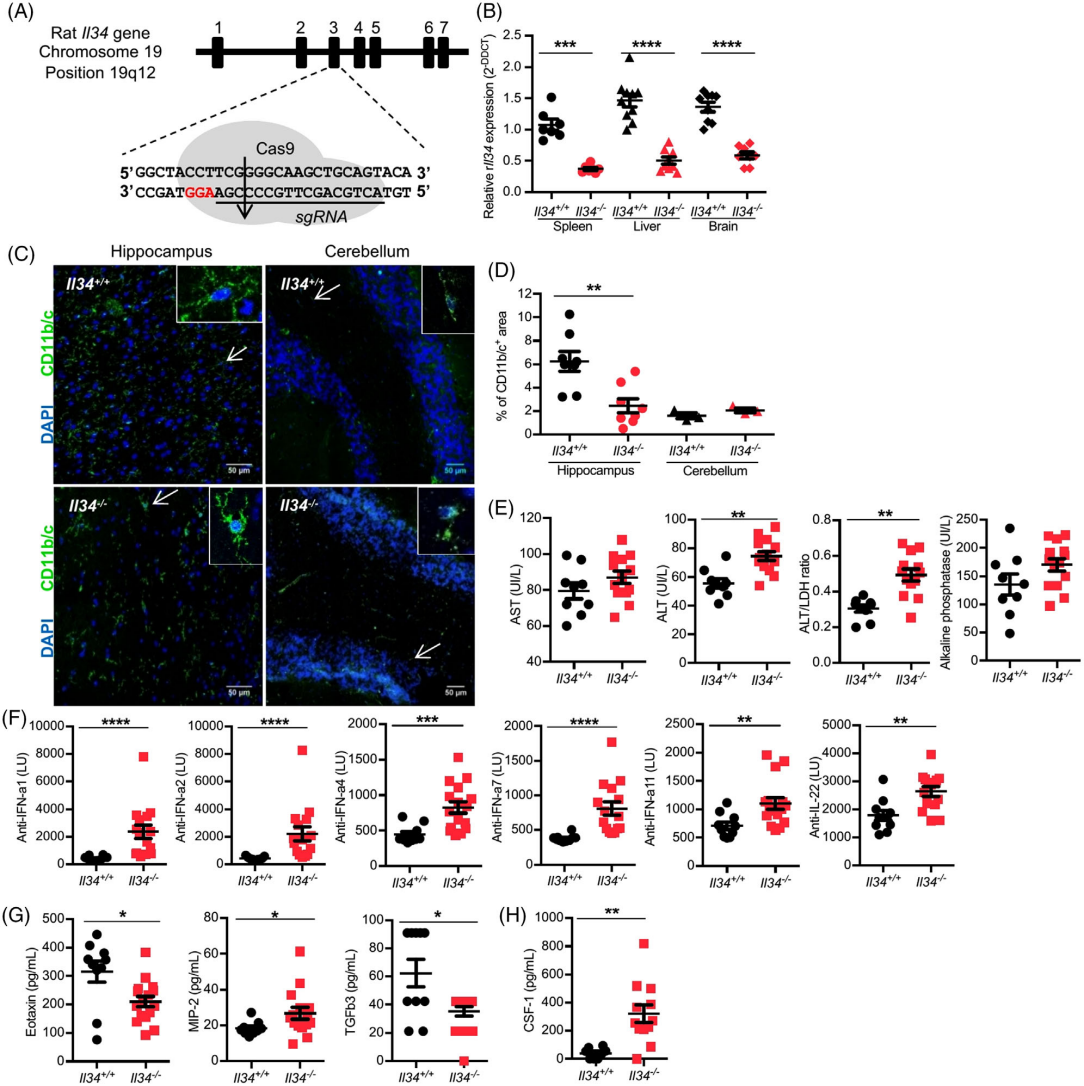
Generation of *Il34* ^−^ *^/^^−^* rats by CRISPR/Cas9 and characterization. (A) Schematic representing the Cas9/sgRNA targeting the exon three of the *Il34* gene inducing a genomic DNA cut (arrow). PAM sequence is in red. (B) *Il34* mRNA expression was assessed by quantitative real‐time PCR in spleen, liver and brain of *Il34^+/+^* (*n* = 7–10) and *Il34* ^−^ *^/^^−^* rats (*n* = 7–9). Results are normalized to *Hprt* and expressed as 2^−ΔΔ^ *^CT^* ± SEM. (C) Confocal microscopy was performed on *Il34* ^+/+^ and *Il34* ^−^ *^/^^−^* rats frozen brains stained with an antibody directed against CD11b/c (green) to identify microglia and DAPI (blue). Arrows indicate a representative stained cell. Original magnification, *×*800. Scale bar 50 μm. (D) Quantification of positive CD11b/c staining areas in *Il34* ^+/+^ and *Il34* ^−^ *^/^^−^* rats’ hippocampus (*Il34* ^+/+^, *n* = 8; *Il34* ^−/−^, *n* = 8) and cerebellum (*Il34* ^+/+^, *n* = 3; *Il34* ^−/−^, *n* = 3) slices. Each dot represents an individual animal and results are expressed as mean ± SEM. (E) Plasma from 4‐month‐old *Il34^+/+^* (*n* = 9) and *Il34* ^−^ *^/^^−^* (*n* = 14) rats was used to quantify AST, ALT, ALT/LDH ratio and alkaline phosphatase. (F) Auto‐antibodies against interferon (IFN)‐α1, α2, α4, α7, α11 and IL‐22 were assessed in sera by luciferase immunoprecipitation systems (LIPS) assay (>6‐month‐old *Il34* ^+/+^ rat, *n* = 10 and *Il34* ^−^ *^/^^−^* rat, *n* = 16). (G) Eotaxin, TGF‐β3 and MIP‐2 protein levels were quantified by Luminex assay in the sera of >6‐month‐old *Il34* ^+/+^ (*n* = 10) and *Il34* ^−^ *^/^^−^* (*n* = 16) rats. (H) CSF‐1 protein was quantified by ELISA in the sera of >6‐month‐old *Il34* ^+/+^ (*n* = 8) versus *Il34* ^−^ *^/^^−^* rats (*n* = 12). Results are expressed as mean ± SEM. Mann–Whitney *U* test, *p < .05, **p < .01, ***p < .001, ****p < .0001

To gain further knowledge on the immunoregulatory role of IL‐34, we generated an *Il34*‐deficient rat model using CRISPR/Cas9 targeting exon 3 of the rat *Il34* gene. The non‐homologous end joining repair mechanism inserted a C base, leading to a STOP codon apparition (Figures 1A and S1A). The founder animal was mated with an *Il34* ^+/+^ animal, the mutation was transmitted to the progeny, and homozygous *Il34* ^−/−^ animals were generated as confirmed by heteroduplex mobility assay on an automated microfluidic chip capillary electrophoresis system and sequencing.^13^, ^14^ In the absence of available anti‐rat IL‐34 antibody, we confirmed the knockout using quantitative RT‐PCR and observed a major decrease of *Il34* mRNA expression in *Il34* ^−/−^ animals compared to littermates *Il34* ^+/+^ animals (Figure 1B). Residual mRNA detected was due to mRNA being produced with an immature STOP codon before rapid degradation. The *Il34* ^−/−^ animals looked visually healthy at steady state with normal appearance (Figure S1B), growth (Figure S1C) and bone mass (Figure S1D,E). However, in accordance with the role of IL‐34 reported for microglia development and maintenance in the mouse,^5^ brain analysis showed decreased microglia (CD11b/c^+^ cells) in the hippocampus, but not the cerebellum of the *Il34* ^−/−^ animals (Figures 1C,D and S1F). Sera analysis showed an increase of ALT and ALT/LDH enzyme levels ratio and a trend for AST and alkaline phosphatase (Figure 1E), suggesting mild liver injury. Interestingly, we also observed a strong increase of auto‐antibodies production against IFN‐α1, −α2, −α4, ‐α7, ‐α11 and anti‐IL‐22 in *Il34* ^−/−^ rats (Figure 1F) (no evidence for anti‐IL‐17A/F Ab), increased inflammatory chemokine MIP‐2 and decreased eosinophil chemotactic proteins (eotaxin), as well as regulatory TGF‐β3 (Figure 1G) (no changes for TGFβ1/2, IL‐1α/β, ‐2, ‐4, ‐5, ‐6, ‐10, ‐12p70, ‐13, ‐17A, G‐CSF, GM‐CSF, TNFα, IFNγ, GROα, MCP‐1, ‐3, MIP‐1α, Rantes and IP‐10; Figure S2A), altogether suggesting auto‐inflammation and an important role for IL‐34 in auto‐antibodies development and cytokines production. Serum levels of immunoglobulin isotypes were normal and antibodies directed against dsDNA were similar to *Il34^+/+^* rats (Figure S2B,C). Histological analysis of organs did not show any evidence of tissue lesions or inflammatory infiltrates (Figure S2D). We investigated whether, as compensatory mechanisms that could mitigate the autoimmune symptoms, CSF‐1 was upregulated and indeed found significantly higher levels of CSF‐1 in the sera of the *Il34^−/−^* animals (>6‐month old) (Figure 1H), suggesting a negative regulation of CSF‐1 by IL‐34.

Altogether, these results demonstrate a role for IL‐34 in rat microglia development and a critical role for regulating auto‐antibody development and cytokine production.

### 2.2 In rat, IL‐34 deficiency leads to a decreased number of CD8^+^ T cells and increased susceptibility to colitis

**FIGURE 2:**
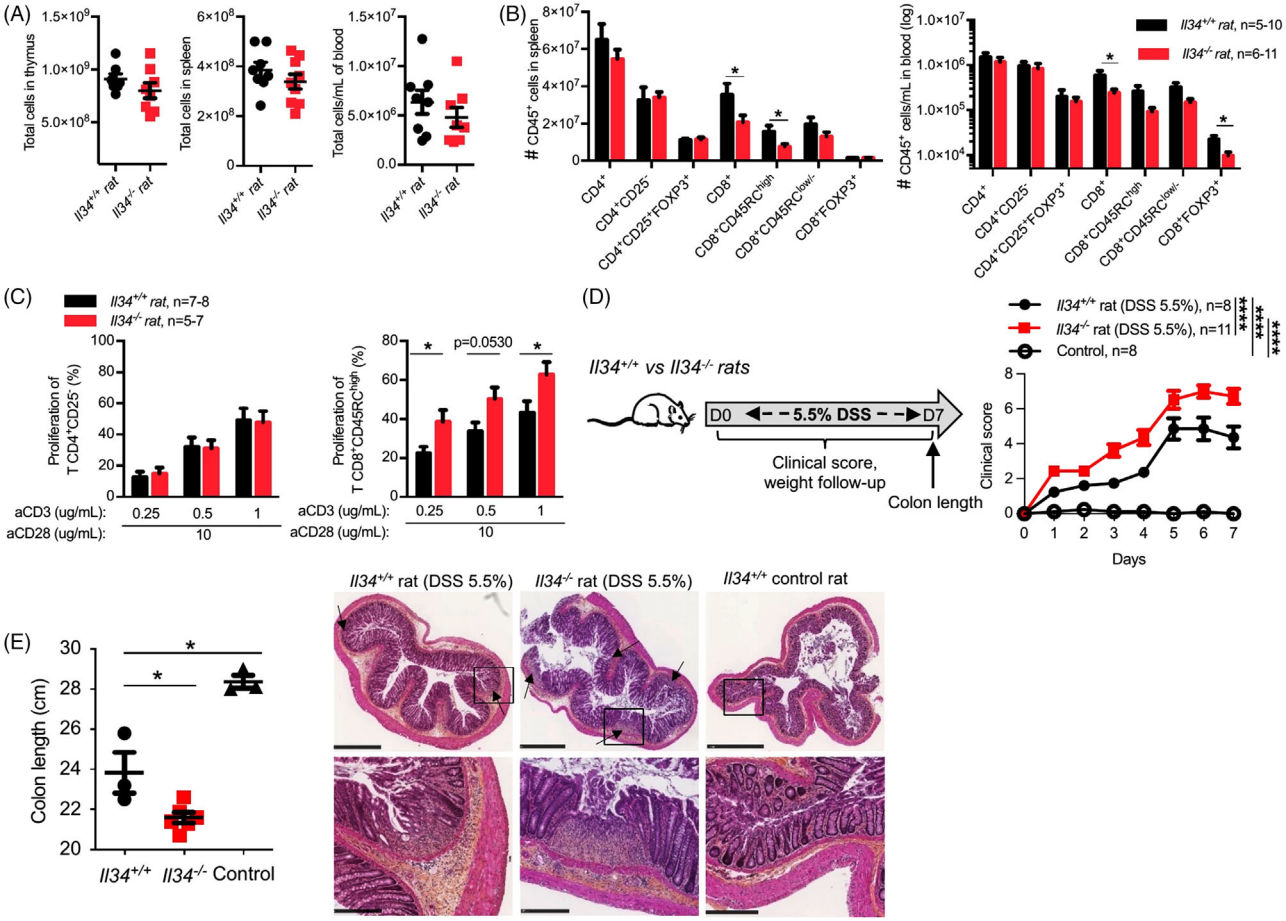
IL‐34 global deficiency increases CD8^+^ T cells proliferation capacity and colitis severity. (A) Absolute numbers of cells were analysed in thymus (2‐month old), spleen and blood (>10‐month old) of *Il34* ^+/+^ (*n* = 5–10) and *Il34* ^−^ *^/^^−^* (*n* = 6–11) rats. (B) Absolute numbers of T cell subsets were analysed in spleen (left) and blood (right) of >10‐month‐old *Il34* ^+/+^ (*n* = 5–10) and *Il34* ^−^ *^/^^−^* (*n* = 6–11) rats using markers described in the Supporting Information section. (C) Effector TCRαβ^+^CD4^+^CD25^−^ (CD4^+^ Teffs) and TCRαβ^+^CD4^−^CD45RC^high^ (CD8^+^ Teffs) from *Il34* ^+/+^ (*n* = 7–8) or *Il34* ^−^ *^/^^−^* (*n* = 5–7) rats were sorted and tested for proliferation capacity over 2‐day culture with an increasing concentration of anti‐CD3 (.25–.5–1 μg/ml) and anti‐CD28 (10 μg/ml) mAbs. Results are represented as mean ± SEM. (D) *Left panel*: Schematic of the acute DSS model: 5.5% of DSS in drinking water was given for 7 days to 9‐week‐old male *Il34* ^+/+^ (*n* = 8) or *Il34* ^−^ *^/^^−^* rats (*n* = 11). *Il34* ^+/+^ control rats were given regular water (*n* = 8). *Right panel*: A clinical score of colitis severity was established and followed every day. (E) At day 7, rats were sacrificed and the colon length measured and H&E stained. Results are represented as mean ± SEM and representative of two independent experiments. Colon sections of *Il34^+/+^* and *Il34^−/−^* rats treated or not with 5.5% of DSS were stained with H&E. Histopathological enlarged images (upper panel, scale bar = 1000 μm) and focused on epithelial injury (lower panel, scale bar = 250 μm) are presented. In colon, crypts rarefaction and/or cell infiltration of the lamina propria and the submucosa were present (indicated by arrows). Data are representative of eight animals in each group. Mann–Whitney *U* test, a two‐way ANOVA with a Bonferroni post‐test for clinical score and log‐rank test for survival analysis, *p < .05, ****p < .0001

We then further analysed the impact of the deficiency on immune cells in mice and rats. Although absolute numbers of cells were slightly decreased in thymus, spleen and blood of *Il34^−/−^* rats (Figure 2A), thymic subpopulations, B cell subsets and myeloid cell subsets in spleen were not modified (Figure S3A–C). In contrast, we observed a significant decrease of CD8^+^ T cells from spleen and blood in *Il34^−/−^* rats versus *Il34^+/+^* rats, but not for CD4^+^ T cells (Figure 2B). In addition, a further analysis of lymphocyte function revealed a higher proliferation capacity of CD8^+^CD45RC^high^ (effector) T cells,^10^, ^15^, ^16^ but not of CD4^+^CD25^−^ effector T cells, in response to a polyclonal stimulation (Figure 2C), altogether suggesting a role for IL‐34 in CD8^+^ T cell number and proliferative capacity.

The phenotype of FOXP3^+^ or FOXP3^−^ CD4^+^ or CD8^+^ T cells did not reveal differences in *Il34^−/−^* rats versus *Il34^+/+^* rats (Figure S3D,E) and we observed similar in vitro proliferation capacity for both CD4^+^ and CD8^+^ Tregs (Figure S3F).

Gaining insights towards the role of IL‐34 in T cell development/function, we next assessed the impact of the global deficiency in an inflammatory environment using the acute DSS colitis model. For 7 days, 5.5% DSS given in drinking water to *Il34^+/+^* and *Il34* ^−/−^ rats resulted in an increased severity at all time points in *Il34^−/−^* animals compared to *Il34^+/+^* animals (Figure 2D). Moreover, analysis of colon length and H&E analysis at day 7 showed that the colon of *Il34^−/−^* rats was shorter than *Il34^+/+^* and control animals and showed histological signs of tissue destruction and leukocyte infiltration (Figure 2E), indicating more inflammation in the colon of *Il34^−/−^* rats. The mean colon length of untreated *Il34^−/−^* rats was 26.63 cm (*n* = 3) similar to control *Il34^+/+^* rats (data not shown). We also observed that DSS‐induced colitis in *Il34^−/−^* rats led to increased levels of pro‐inflammatory cytokines *Il6*, *Il1b*, *Il22* and a trend for an increase for *Il17a*, all involved in gut inflammation, whereas *Ifng* was decreased (Figure S4A). However, we observed no significant differences between *Il34^+/+^* and *Il34^−/−^* rats. Altogether, our data demonstrate that the lack of IL‐34 upon DSS‐induced colitis increases its severity.

### 2.3 IL‐34 deficiency leads to defective suppressive function of CD4^+^ Tregs in vivo

**FIGURE 3:**
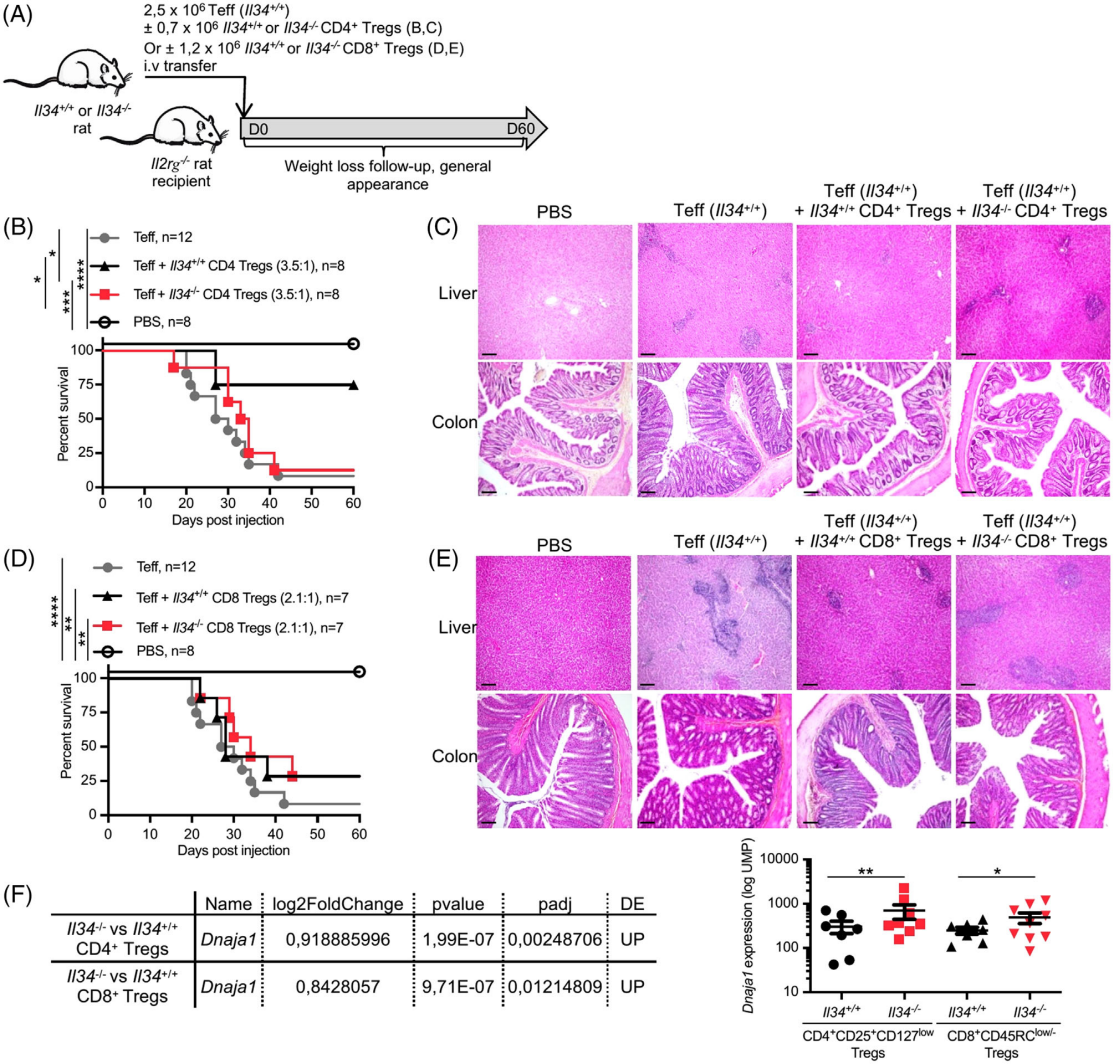
*Il34^−^^/^^−^* CD4 Tregs fail to protect from the wasting disease. (A) Schematic showing the wasting disease model induction. (B) Six‐week‐old *Il2rg* ^−^ *^/^^−^* rats were injected i.v. with 2.5 × 10^6^ TCRαβ^+^CD4^+^CD45RC^high^ Teffs from *Il34^+/+^* rats (Teffs; *n* = 12) in association or not with TCRαβ^+^CD4^+^CD25^+^CD127^low^ Tregs (CD4^+^ Tregs; 3.5:1 Teffs:Tregs ratio) from *Il34^+/+^* (*n* = 8) or *Il34^−/−^* (*n* = 8) rats in seven independent experiments. (C) Liver and colon sections of injected *Il2rg* ^−^ *^/^^−^* rats were stained with haematoxylin eosin saffron (HES) (original magnification *×*10, scale bar 20 μm). Data are representative of six animals in each group. (D) Six‐week‐old *Il2rg* ^−^ *^/^^−^* rats were injected i.v. with TCRαβ^+^CD4^+^CD45RC^high^ Teffs from *Il34^+/+^* rats (Teffs) in association with or not with TCRαβ^+^CD4^−^CD45RC^low/−^ Tregs (CD8^+^ Tregs; 2.1:1 Teffs:Tregs ratio) from *Il34^+/+^* (*n* = 7) or *Il34^−/−^* (*n* = 7) rats in five independent experiments. (E) Liver and colon sections of injected *Il2rg* ^−^ *^/^^−^* rats were stained with HES (original magnification *×*10). Data are representative of six animals in each group. (F) (left) 3′ DGE‐RNA sequencing analysis was performed on sorted and non‐stimulated TCRαβ^+^CD4^+^CD25^+^CD127^low^ Tregs (CD4^+^ Tregs) and TCRαβ^+^CD4^−^CD45RC^low/−^ Tregs (CD8^+^ Tregs) from 8–10‐week‐old *Il34^+/+^* (*n* = 7) and *Il34^−/−^* rats (*n* = 8–9). The table recapitulates the results of *Dnaja1*, the only gene differentially expressed between *Il34^−/−^* versus *Il34^+/+^* CD4^+^ Tregs and *Il34^−/−^* versus *Il34^+/+^* CD8^+^ Tregs. (Right) Transcript levels of *Dnaja1* (UPM) log scale in CD4^+^ and CD8^+^ Treg in *Il34^+/+^* or *Il34^−/−^* rats. Mann–Whitney *U* test, a two‐way ANOVA with a Bonferroni post‐test for clinical score and log‐rank test for survival analysis, *p < .05, **p < .01, ***p < .001, ****p < .0001

As we previously showed that around 50% of FOXP3^+^ CD4^+^ and CD8^+^ Tregs express IL‐34,^6^ we then tested CD4^+^CD25^+^ (CD4^+^ Tregs) and CD8^+^CD45RC^low/−^ (CD8^+^ Tregs) Tregs from *Il34^−/−^* rats for their capacity to control in vivo wasting disease by i.v. transfer of T CD4^+^CD45RC^high^ effector cells in *Il2rg^−/−^* rats (Figure 3A). *Il2rg^−/−^* rats are immunodeficient, and they lack or have greatly reduced numbers of T, NK and B cells.^17^ The use of *Il2rg^−/−^* rats reproduces a classical model of colitis in both immunodeficient mice (nude or different mutations in *Rag* or *Il2rg* genes) and rats (nude). In both species, an immunodeficient recipient receiving CD4^+^CD45RB^high^ (mouse) or CD4^+^CD45RC^high^ (rat) cells causes a wasting disease with intestinal and liver lesions, among other organs.^17^ Strikingly, *Il34^−/−^* CD4^+^ Tregs were unable to control the development of wasting disease in *Il2rg^−/−^* animals and death occurred in a similar kinetic rather than without CD4^+^ Tregs, in contrast to CD4^+^ Tregs from *Il34^+/+^* rats that efficiently controlled wasting disease development (Figure 3B), demonstrating the critical role of IL‐34 in CD4^+^ Tregs–mediated suppressive activity. Histological analysis of the liver and colon from all groups confirmed the cell infiltration and tissue lesions in *Il2rg^−/−^* animals injected with CD4^+^ Teff cells without or with CD4^+^ Tregs from *Il34^−/−^* animals, in contrast to animals injected with CD4^+^ Treg from *Il34^+/+^* animals (Figure 3C). Although we did not observe a protective role of CD8^+^ Tregs in this model for both *Il34^+/+^* and *Il34^−/−^* CD8^+^ Tregs (Figure 3D,E), we observed decreased infiltration in liver and colon in animals injected with CD8^+^ Tregs from *Il34^+/+^* animals compared to animals injected with CD4^+^ Teff cells with or without CD8^+^ Tregs from *Il34^−/−^* animals (Figure 3E), suggesting that *Il34^−/−^* CD8^+^ Tregs were less efficient.

To understand how IL‐34 deficiency had modified the function of both CD4^+^ and CD8^+^ Tregs in *Il34^−/−^* versus *Il34^+/+^* rats, we analysed their transcriptome using differential gene expression (DGE)‐RNAseq. Intriguingly, the transcriptomic landscape of CD4^+^ and CD8^+^ Tregs in *Il34^−/−^* rats only significantly differed for one gene, *Dnaja1*, a co‐chaperone of heat shock proteins (HSPs),^18^ identically increased in CD4^+^ and CD8^+^ Tregs from *Il34^−/−^* rats versus CD4^+^ and CD8^+^ Tregs from *Il34^+/+^* rats (Figure 3F). Thus, although the role of the increased mRNA expression of *Dnaja1* needs to be explored in future work, the major defect in suppressive capacity observed for Tregs from *Il34^−/−^* rats is likely not due to a major effect on Tregs direct functions but rather on downstream effects of IL‐34 produced by Tregs, mainly through induction of tolerogenic macrophages.^4^, ^6^, ^9^

### 2.4 *Il34^−/−^* mice are more prone to autoimmunity

**FIGURE 4:**
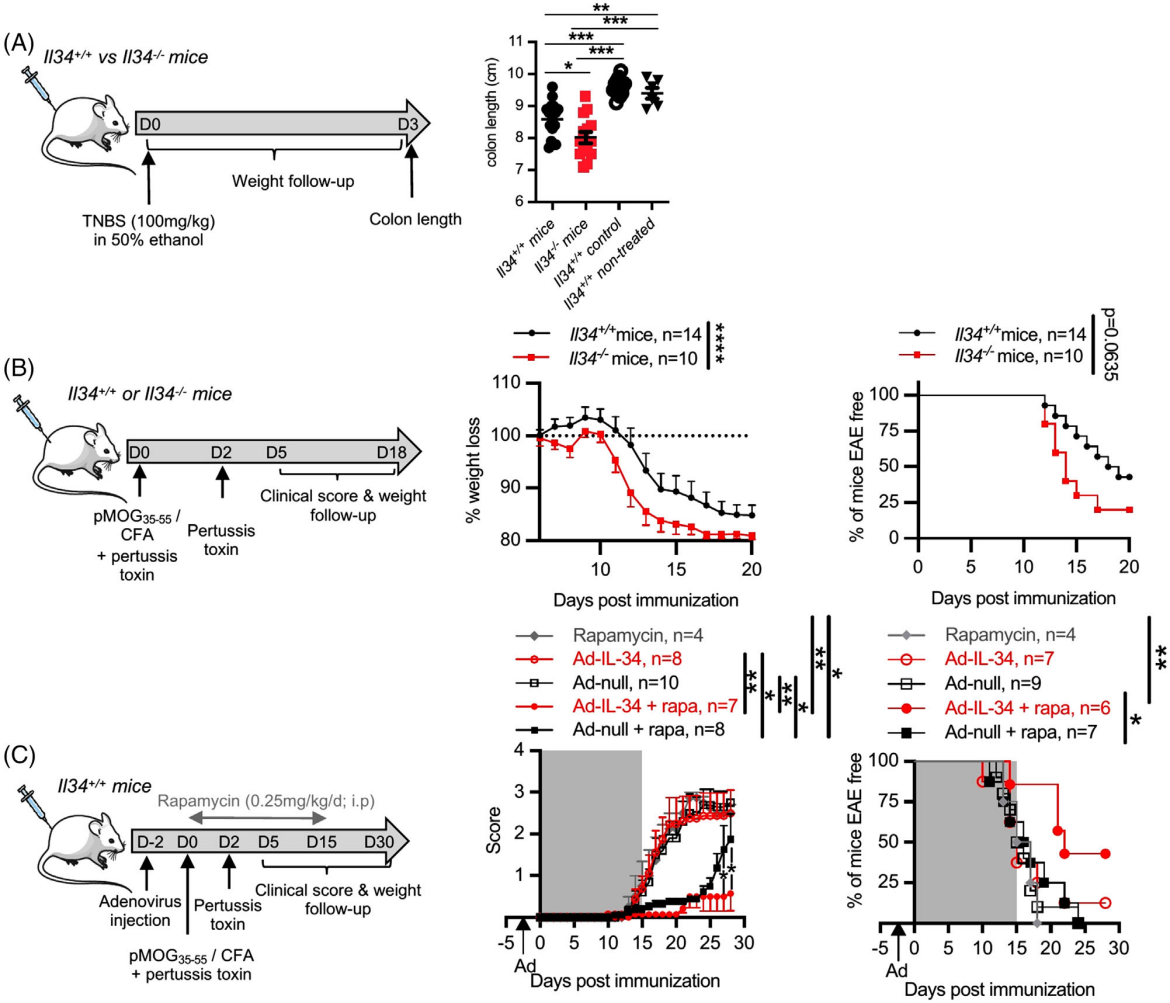
*Il34^−^^/^^−^* mice are more susceptible to autoimmune diseases. (A) Schematic showing the TNBS‐induced colitis. The model was performed on *Il34^+/+^* versus *Il34^−/−^* mice (*n* = 11–13) via the intrarectal administration of TNBS (100 mg/kg) in 50% ethanol. Control *Il34^+/+^* mice were injected with 50% ethanol alone or not treated. Mice colon length was measured at day 3 post‐injection. Each dot represents an animal. Results are represented as mean ± SEM. (B) Schematic showing the MOG‐induced experimental autoimmune encephalomyelitis (EAE) model in mice. Adult *Il34* ^+/+^ (*n* = 14) and *Il34* ^−^ *^/^^−^* (*n* = 10) C57BL/6J mice of 8‐week old were immunized with the peptide MOG_35–55_ and mycobacterium for EAE induction. The development of the disease was followed by a daily weight and scoring assessment. (C) Schematic showing the IL‐34 overexpression in the MOG‐induced EAE model. Adult *Il34^+/+^* mice of 8‐week old were i.v. injected with an adenovirus coding for mouse IL‐34 (Ad‐IL‐34) or a control (Ad‐null) 2 days before inducing the EAE. Rapamycin was administered or not every day for 15 days at a dose of .25 mg/kg/d (i.p. injection; grey area). The development of the disease was followed by a daily weight and scoring assessment. Results are represented as mean ± SEM. Mann–Whitney *U* test or two‐way ANOVA and a Bonferroni posttest for the score analyses. *p < .05; **p < .01, ***p < .001, ****p < .0001

To take advantage of the *Il34^−/−^* mouse model,^12^ we then assessed susceptibility to autoimmune diseases in *Il34^−/−^* mice in models of TNBS‐induced colitis (Figure 4A) and experimental autoimmune encephalomyelitis (EAE) triggered by MOG_p35–55_ (Figure 4B).^12^ In the TNBS‐induced colitis model, we observed a shorter colon length at day 3 in *Il34^−/−^* mice compared to *Il34^+/+^* and *Il34^+/+^* control mice (Figure 4A), indicating more severe inflammation; however, we did not observe significant differences in weight loss (Figure S4B). The mean colon length of untreated *Il34^−/−^* mice was 9.43 ± .28 cm (*n* = 3) similar to control *Il34^+/+^* mice (9.29 ± .17 cm) (data not shown). We next investigated the impact of IL‐34 deficiency in the EAE model as IL‐34 could have a protective role through Treg maintenance and microglia development.^6^, ^12^, ^19^ In the EAE model, the *Il34^−/−^* mice developed EAE faster and more severely and most *Il34^−/−^* mice died by day 20 as shown by the survival curve (Figure 4B), in contrast to the *Il34^+/+^* animals. In addition, *Il34^−/−^* mice lost more weight than the controls; however, the overall clinical score was not significantly different (Figure S4C). When we assessed the therapeutic potential of IL‐34 therapy in autoimmunity in the EAE model, we observed that the injection of an adenovirus coding for murine IL‐34 in association with a suboptimal dose of rapamycin led to a delayed EAE development compared to the control groups, that is, null adenovirus associated or not with rapamycin (Figure 4C, left). We observed that the mice treated with Ad‐null + rapamycin developed signs of EAE faster than the group Ad‐IL34 + rapamycin; however, the score remains low until day 20–25 before increasing, whereas the group treated with Ad‐IL34 remained stable (Figure 4C, right).

Thus, altogether, IL‐34 deficiency in mice leads to an unstable immune phenotype, with increase susceptible to autoimmunity and inflammation in a specific environment. Moreover, supplementing IL‐34 is beneficial in neurodegenerative autoimmune diseases in mice.

### 2.5 hIL‐34 protein infusion with a suboptimal dose of rapamycin demonstrates regulatory therapeutic properties

**FIGURE 5:**
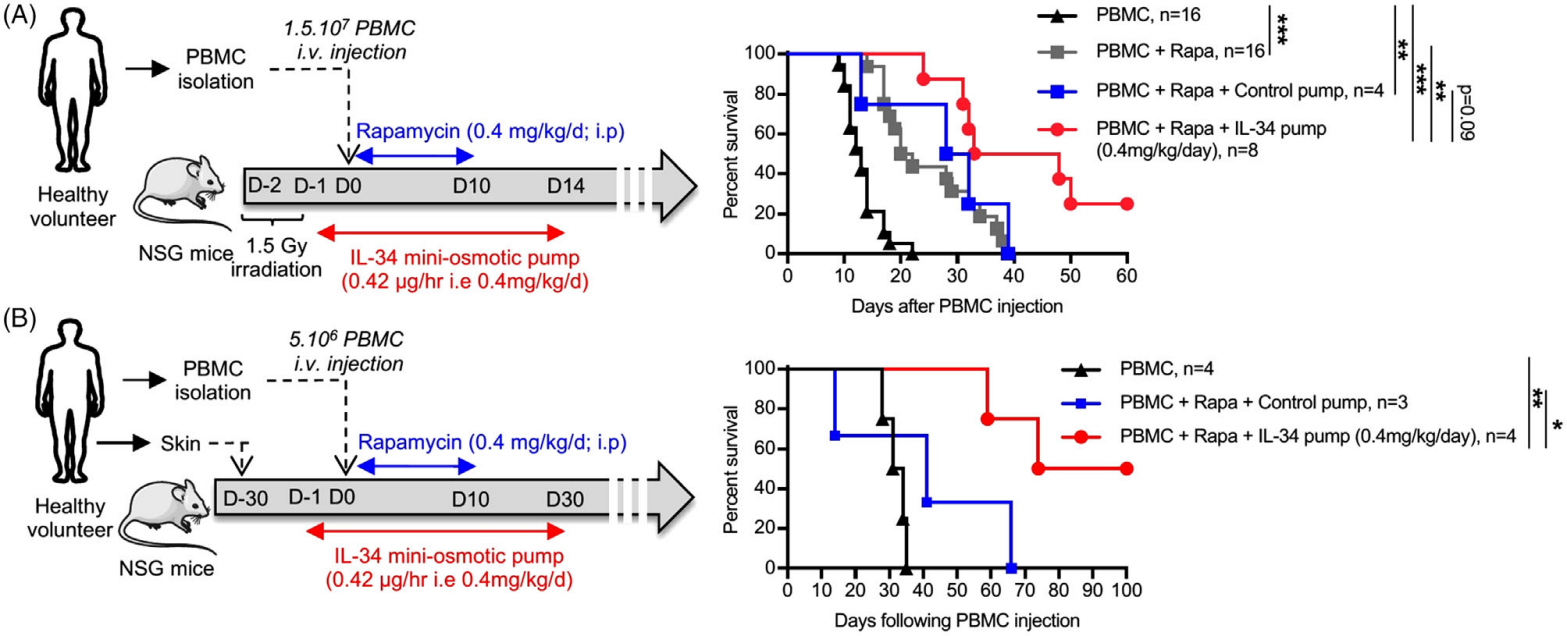
Human IL‐34 recombinant protein administration delays graft‐versus‐host‐disease (GVHD) as well as skin allograft rejection in humanized NSG mice. The therapeutic potential of IL‐34 was tested in (A) GVHD and (B) human skin graft allogeneic rejection models in humanized immunodeficient NSG mice. (A) IL‐34 was administered with a mini‐osmotic pump delivering a constant rate for 14 days (.42 μg/h) with or without rapamycin (.4 mg/kg/d for 10 days). (A) For GVHD, 1.5 × 10^7^ fresh PBMCs were i.v. injected in 1.5‐Gy irradiated 8–12‐week‐old NOD/SCID/*IL2rg^−/−^* (NSG) mice, and the survival of mice was measured by weight loss. (B) For skin allograft rejection, 5 × 10^6^ fresh PBMCs were i.v. injected in mice grafted with human skin 4 weeks before. Graft survival was scored on macroscopic signs of rejection from 0 to 5 and considered rejected at a score of 3. Log‐rank tests for survival analysis. *p > .05, **p < .01 and ***p < .001

Our data so far present a critical role for IL‐34 in Treg‐mediated suppression and although we previously showed that IL‐34 played a role in a transplantation model in rats and mice and that IL‐34 inhibited human Teff cells in vitro,^6^, ^9^ the question of whether IL‐34 has the capacity to inhibit transplant rejection and induce transplant tolerance in an immune response by human cells still remains. Thus, we used transplantation models in immune‐humanized immunodeficient NSG mice (Figure 5A,B) in which we administered recombinant human IL‐34 (rhIL‐34) protein using mini‐osmotic pumps delivering a constant rate of protein and assessed xenogeneic GVHD or human skin transplant rejection. We previously demonstrated that the administration of IL‐34 alone (.8 mg/kg/2 d) did not delay GVHD development compared to PBMC alone;^9^ thus, we used IL‐34 together with a suboptimal dose of rapamycin. Administration for 14 days of rhIL‐34 protein in association with a suboptimal dose of rapamycin for 10 days resulted in significantly delayed GVHD development with an induction of long‐term survival in 25% of the recipients (Figure 5A) and a significant induction of long‐term skin graft tolerance in 50% of the recipients (Figure 5B), demonstrating the potential of this therapeutic strategy. Analysis of the proportion of hCD45^+^ cells showed a decrease in animals treated with rapamycin alone versus control animals and equal inhibition in animals co‐treated with rapamycin and rhIL‐34 in the acute GHVD model (Figure S5A), whereas in the human skin transplantation model, the proportion of hCD45^+^ cells was not modified (Figure S5B). Cell subpopulation frequencies (CD3^+^, CD14^+^, CD19^+^, CD56^+^), CD4^+^ and CD8^+^ Teff/Tregs cells were also not impacted by IL‐34 treatment in both models (Figure S5C,D). These results can be explained by the inhibition of T effector development and/or function rather a direct inhibition of human lymphocyte proliferation by rapamycin and IL‐34. Whether this is due to an increase in Tregs function needs further studies.

### 2.6 Pre‐transplantation human IL‐34 serum levels, but not of CSF‐1, is a prognostic maker of a higher rejection‐free episode

**FIGURE 6:**
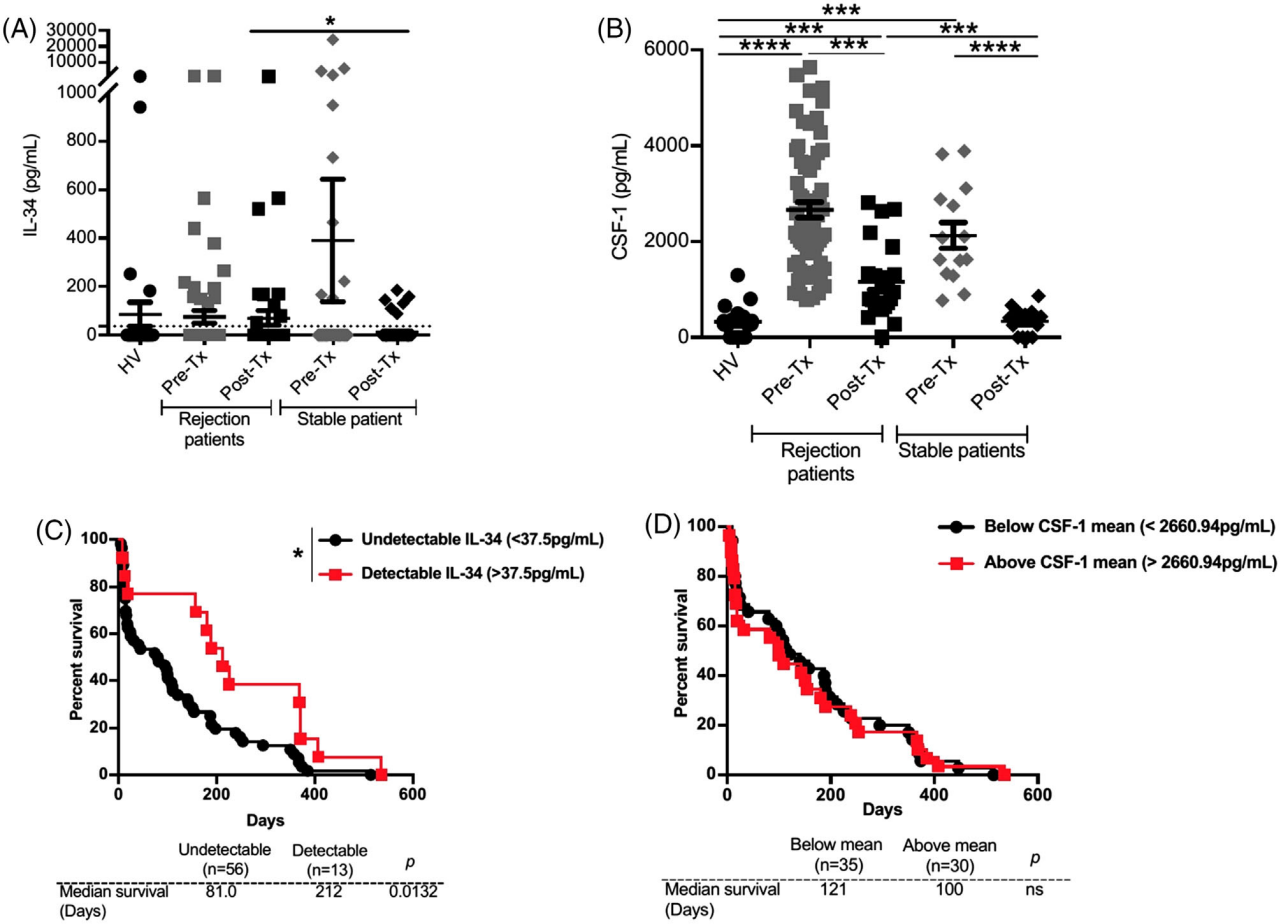
Pre‐transplantation IL‐34 levels as a marker of higher rejection‐free episodes in human kidney transplant patients. (A) IL‐34 and (B) CSF‐1 were quantified in the serum of healthy volunteers (*n* = 30 and *n* = 20, respectively) and in patients before (Pre‐Tx) and after (Post‐Tx) transplantation with a stable graft function or having had ≥1 episode of acute rejection within the 18 months following Tx (see Table S3 for clinical characteristics; pre‐Tx: rejection patients *n* = 71 and *n* = 65, stable patients *n* = 101 and *n* = 14; post‐Tx: rejection patients *n* = 42 and *n* = 22, stable patients *n* = 71 and *n* = 15 for IL‐34 and CSF‐1 serum levels respectively). (C and D) Graft survival of patients with an acute rejection episode occurrence according to pre‐Tx IL‐34 detectability (undetectable: ≤37.5 vs. detectable: ≥37.5 pg/ml) (C) or CSF‐1 mean expression (below vs. above 2660.94 pg/ml) (D). Mann–Whitney *U* test and log‐rank tests for survival analysis. *p > .05, **p < .01, ***p < .001 and ****p < .0001

We finally assessed the potential use of IL‐34 as a biomarker in organ transplantation compared to CSF‐1 using serum samples from the DIVAT cohort biocollection (Table S3). We quantified IL‐34 and CSF‐1 by ELISA before (Pre‐Tx) and within 18 months after kidney transplantation (Post‐Tx). Moreover, the outcome of the graft is known during the 18 months, that is, stable graft function, free of rejection episodes or with episodes of acute rejection, was used to identify stable patients versus rejection patients (Figure 6A–D). We first determined the detectability of IL‐34 based on the detection threshold: 37.5 pg/ml, that is, the lowest concentration of the standard curve provided by the manufacturer in the sera of patients. IL‐34 serum levels were detectable (≥37.5 pg/ml) in around 22% of patients in the different groups (Figure S6A) with increased levels in patients who had experienced a rejection compared to stable patients at the post‐transplantation period (Figures 6A and S6D). In contrast, CSF‐1 was detectable in most individuals and levels were higher in pre‐transplantation compared to post‐transplantation as well as in healthy volunteers. CSF‐1 levels were also higher in post‐transplantation with rejection versus stable patients (Figure 6B). We further analysed the correlation between IL‐34/CSF‐1 and the clinical outcome to determine the possibility of using IL‐34 or CSF‐1 as a prognosis biomarker. We split patients with rejection episodes in two groups based on IL‐34 detectability (≤37.5 pg/ml versus ≥37.5 pg/ml) (FigureS 6C and S6A) or CSF‐1 mean expression for all patients (≤2660.94 pg/ml vs. ≥2660.94 pg/ml) (Figure 6D) in serum before transplantation and analysed the graft survival period before acute rejection episode occurrence. Interestingly and unlike CSF‐1, the proportion of patients without rejection episodes in patients with IL‐34 levels ≥37.5 pg/ml before transplantation was significantly higher than the one of patients with IL‐34 below this level. This statistical correlation was specific when measured before transplantation as IL‐34 levels in serum after transplantation did not predict graft outcome (Figure S6B,C). Our results thus suggest that IL‐34 can act as a prognosis biomarker before transplantation.

## 3 DISCUSSION

Here, we demonstrated IL‐34 as a critical actor of Treg‐mediated suppression, playing an important role in homeostasis and control of immune responses. IL‐34 expression in the T cell lineage seems to be restricted to FOXP3^+^ CD4^+^ and CD8^+^ Tregs^6^, ^9^ acting as a significant suppressor component of their activity. IL‐34 is also expressed by other cell subsets such as neurons playing as well a protective role for microglia.^20^, ^21^

At steady state, the overall phenotype of *Il34^−/−^* rats remained similar to the one of *Il34^−/−^* mice^12^, ^19^ with no evident developmental or pathological signs with the exception of a decrease in microglia. There has not been a thorough description of the distribution and function of immune cell subsets in *Il34^−/−^* mice or on the response to inflammatory/autoimmune stimuli, as described here for *Il34^−/−^* animals. The increased expression of auto‐antibodies in *Il34^−/−^* rats is intriguing suggesting a role for IL‐34, the regulation of antibody production. CD138, a co‐receptor of IL‐34, is expressed by plasma cells and binding of IL‐34 to CD138 stimulates migration but does not transduce signalling in the absence of CSF‐1R. Plasma cells are negative for CSF‐1R; however, the expression of PTPz has not been reported on plasma cells; thus, IL‐34 could potentially impact plasma cells through CD138. These auto‐antibodies include anti‐IFN‐I antibodies that in addition to the role of pro‐inflammatory monocytes/macrophages in the cytokine storm leading to severe acute respiratory distress syndrome and multi‐organ pathology observed in SARS‐CoV‐2, infection could suggest a role for IL‐34 in COVID‐19 patients^22^, ^23^ and a link between IL‐34 and AIRE in T cell education in the thymus as high titres of IFNα auto‐antibodies are found in APECED patients^14^, ^24^ and *Aire^−/−^* rats^25^ which harbour inborn errors in AIRE, which is responsible for thymic expression of ectopic self‐antigens and is thus responsible for negative T cell selection and Treg education in the thymus. The presence of anti‐IFNα and anti‐IL‐22 auto‐antibodies could also be linked to the mild liver injury observed in *Il34^−/−^* rats.^26^ Further investigation on the impact of IL‐34‐deficiency in the gut‐liver axis and whether IL‐34 deficiency may exacerbate the complications observed in caecal ligation and puncture models would be interesting.^27^

IL‐34 deficiency in rats led to a mild immune phenotype in vivo at resting state, probably due to CSF‐1 overexpression that may partially compensate IL‐34 deficiency in their common overlapping functions, particularly in monocyte and macrophage differentiation. These results suggest a negative regulation of CSF‐1 by IL‐34, complementary to the positive regulation of CSF‐1 on IL‐34 previously described.^28^ Despite that CSF‐1 could replace activities of IL‐34, we still observed some features of a disturbed immune system and differences on the CD8^+^ T cells lineage distribution pattern. However, upon challenges, we revealed an increased susceptibility to colitis. Even though we did not find any perturbation in myeloid distribution in the gut at steady state (data not shown), one explanation could be that the inflammatory environment could not be counterbalanced by the action of IL‐34 on macrophage polarization leading to a greater severity in deficient animals. In the EAE model, the action of IL‐34 can be mediated either through the development of microglia^5^ or through Treg activity both in the CNS and periphery. We did find accelerated EAE development in *Il34^−/−^* mice compared to *Il34^+/+^* mice as by day 15 almost all *Il34^−/−^* mice had lost >20% weight, but we did not find significant differences in the score. Overexpression of IL‐34 was beneficial and significantly delayed EAE development showing a therapeutic potential of IL‐34 in the initial phases of inflammatory CNS diseases. In this model, we used a suboptimal dose of rapamycin, which found that, compared to higher doses given alone, it has a minor effect on the clinical course of the disease, as we previously showed that there is a synergistic effect of rapamycin and IL‐34 on a model of acute graft rejection in rats^6^ and to benefit from the promoting effect of rapamycin on Tregs. Rapamycin has been used in the EAE and transplantation models in this manuscript and although it is not the main treatment in the corresponding disease in humans, multiple sclerosis and aGVHD, there have been studies showing that its use can be of interest. Rapamycin has been used to alleviate EAE in mice^29^, ^30^ and has been very recently proposed as a treatment to screen for new therapies in multiple sclerosis.^31^ More importantly, rapamycin has been used in multiple sclerosis patients^32^ (https://clinicaltrials.gov/ct2/show/NCT00095329). In aGVHD, rapamycin has been used in recent translational work, which has been done in the humanized NSG model^33^ as well as in clinical trials.^34^, ^35^ When we specifically studied the impact of the deficiency in Treg subsets, we revealed a clear defect in the IL‐34‐deficient CD4^+^ Tregs‐suppressive function where they fail to protect from the wasting disease. This could be due to an insufficient differentiation of M2‐like macrophages. This is reminiscent of our observations that IL‐34 induces immune tolerance in a model of organ transplantation through M2 macrophages and downstream induction of Treg,^9^ creating a positive regulatory loop. In in vitro suppressive assay, we could not find a significant defect of suppressive capacity of CD4^+^ and CD8^+^ Tregs, but this might be due to the setting in which this assay is performed using irradiated allogeneic T cells depleted‐splenocytes and analysis after 6 days incubation (data not shown). Interestingly, transcriptomic analysis of T cells revealed the upregulation only of *Dnaja1* in both *Il34^−/−^* CD4^+^ and CD8^+^ Tregs compared to *Il34^+/+^* rats. *Dnaja1* encodes for a protein belonging to the HSP40 acting as an HSP70 co‐chaperone. One report has demonstrated a regulatory role of Dnaja1 where it inhibits T cell proliferation and induces IL‐10 production by PBMC in rheumatoid arthritis patients.^36^ Further work needs to be done in order to comprehend the link between IL‐34 and Dnaja1; however, this overall shows that IL‐34 has overall no impact on Treg transcriptomic profile itself, and this was confirmed by analysing the transcriptomic profile of Tregs following a short stimulation (data not shown). Thus, the loss of function we observe in the wasting disease model can be exclusively attributed to the lack of IL‐34 production by Tregs.

IL‐34 upregulation has been associated with certain but not all autoimmune diseases. However, the exact contribution of IL‐34 in these diseases has not been elucidated as IL‐34 upregulation could be a compensatory tolerogenic mechanism, thus a consequence rather than a cause.^1^, ^2^ Moreover, it is important to take into consideration that although IL‐34 and CSF‐1 share the CSF‐1R that could explain results through M2 cells, IL‐34 has two other receptors or co‐receptors, PTPzeta and CD138, expressed on other cell types, which could also participate in its final effects.^2^, ^5^ IL‐34 was shown to delay solid organ graft rejection in animal models and associated with a delayed liver rejection in human.^6^, ^11^ Although in our previous work we investigated the potential of ex vivo expanded Treg in the presence of IL‐34 to inhibit GVHD in humanized NSG mice,^9^ here, we demonstrate the potential of using directly IL‐34 as an immunotherapy in vivo in human. Indeed, the delay in skin rejection and GVHD in the NSG immune humanized models of the present manuscript are the first ones showing a potent suppressive effect on human immune responses in vivo. As monocytes in NSG mice are only detected in the first few days post‐hPBMC injection and rapamycin is needed in this context and in the allotransplantation model in rat,^6^ we believe, in accordance with previous results,^9^ that IL‐34 first acts on monocytes to differentiate them in M2‐like macrophages which will allow to expand Tregs, creating a regulatory environment.

Along this line of inhibition of allogeneic immune responses by IL‐34 and prolongation of the rejection‐free post‐transplantation period in vivo upon overexpression, the detection of IL‐34 in the sera of patients before kidney transplantation also defined patients who have a higher probability of having a longer rejection‐free post‐transplantation period. Thus, and unlike CSF‐1, detectable IL‐34 levels in the serum before transplantation could serve as a biomarker of better kidney transplant outcome. Still, this finding needs to be confirmed in a larger cohort and confronted to other cohorts of transplanted patients, and a correlation with other biological and clinical marker would be interesting. However, after transplantation, there was no correlation between rejection and stable patients, but this could be due to the impact of immunosuppressive drugs taken by the patients.

Overall, we show that IL‐34 is crucial to CD4^+^ Tregs suppressive function as well as its deficiency led to increased susceptibility in some autoimmune processes. In human, the administration of IL‐34 represents a new therapeutic strategy for the treatment of autoimmune diseases, GVHD and transplant rejection.

## 4 METHODS

### 4.1 Animals

Sprague‐Dawley (CD) rats were purchased from Charles River Laboratories, France (L'Arbresle, France) and C57Bl/6 mice from Janvier Labs (Le Genest‐Saint‐Isle, France). The 8–12‐week‐old NOD/SCID/*Il2rg^−/−^* (NSG) mice were bred in our own animal facilities in SPF conditions. *Il34* ^LacZ/LacZ^ mice were kindly provided by Marco Colonna (Washington University, St. Louis, MO).^12^ *Il2rg^−/−^* rats were kindly provided by TRIP Platform (Nantes, France) and have been previously described.^37^ The animals were housed in a controlled environment (temperature 21 ± 1°C, 12‐h light/dark cycle).

### 4.2 Generation and genotyping of *Il34* ^−/−^ SPD rats

rIl34‐sgRNA4 (TGTACTGCAGCTTGCCCCGA) was designed and generated as previously described.^38^ Prepubescent females (4–5‐week old) were injected with 25‐IU pregnant mare serum gonadotropin (Intervet) and followed 48 h later with 30‐IU human chorionic gonadotropin (Centravet, Plancoet, France) before breeding.^38^ Fertilized one‐cell stage embryos were collected for subsequent microinjection using a previously published procedure.^38^, ^39^

The sgRNA to target rat *Il34* (TGTACTGCAGCTTGCCCCGA) was in silico defined using the CRISPOR software.^40^ The sgRNA chosen had a cutting frequency determination (CFD) score of 1, whereas all the other potential off‐target had a weak CFD score *I* < .4 with at least 3–4 mismatches and these potential off‐target sequences were situated in intergenic or intronic sequences in chromosomes others than *Il34*. sgRNA (10 ng/μl) and Cas9 mRNA (50 ng/μl)^38^ were co‐microinjected into the cytoplasm and nucleus of one‐cell‐stage fertilized embryos. Surviving embryos were implanted in the oviduct of pseudo‐pregnant females (.5 dpc) and allowed to develop until birth. For genotyping of animals’ ear biopsy specimens from 8‐ to 10‐d‐old rat pups were digested in 250 μl of tissue digestion buffer (Tris‐HCl .1 mol/L [pH 8.3], EDTA 5 mmol/L, SDS .2%, NaCl .2 mol/L, proteinase K 100 μg/ml) at 56°C overnight. PCR amplification was performed on diluted lysis product (1:20 dilution) and 25 μl of PCR reaction mix according to the manufacturer instruction (Herculase II Fusion DNA Polymerase, Agilent Technologies, Les Ulis, France) using the following PCR primers: forward 5′‐AGGTGGAGTACAGACACAGT‐3′ and reverse 5′‐AGATAAGAGGTGGGAGTGAGC‐3′. The following amplification program was used: 1 cycle at 95°C for 5 min, 1 cycle at 62°C for 2 min, 35 cycles at 72°C for 30 s, 95°C for 10 s, and 60°C for 10 s, followed by 1 cycle at 72°C for 3 min using a Veriti Thermal Cycler (Applied Biosystems, Foster City, CA, USA). The PCR products were analysed by heteroduplex mobility assay using microfluidic capillary electrophoresis system caliper LabChip GX (PerkinElmer, Villebon‐sur‐Yvette, France). The homozygous *Il34^−/−^* specimens were identified from *Il34^+/+^* littermate as previously described.^13^

### 4.3 Bone density

Femurs of *Il34^+/+^* and *Il34^−/−^* rats were fixed 24 h in PFA 4%, then trabercular bone mineral density and cortical tissue mineral density were analysed using the high‐resolution SkyScan‐1076 X‐ray microCT system (SkyScan, Kartuizersweg, Belgium).

### 4.4 ELISA

Rat CSF‐1 was quantified using a rat M‐CSF ELISA (MyBioSource, San Diego, CA, USA). Human CSF‐1 and IL‐34 human serum levels were quantified using ELISA kits (both R&D systems, Mountain View, CA, USA), according to manufacturer's instructions. Anti‐dsDNA antibodies were quantified by coating 100 μg/ml of salmon sperm DNA (Invitrogen) in a 96‐well flat‐bottom plate (Thermo Fisher Scientific) in PBS overnight at 4°C. After five washes in PBS‐Tween 20 .05%, saturation with PBS‐BSA 5%‐FCS 2% was performed for 1.5 h at 37°C. Sera from *Il34^+/+^* or *Il34^−/−^* rats were serially diluted in PBS‐BSA 1% (1/2, 1/20, 1/200 and 1/2000) and incubated for 1 h at 37°C. After 10 washes with PBS‐Tween 20 .05%, an HRP‐conjugated goat anti‐rat IgG (heavy and light) (Jackson ImmunoResearch) was used as secondary Ab (1 h, 37°C). After 10 washes with PBS‐Tween 20 .05%, the reaction was visualized by the addition of chromogenic substrate (TMB, BD Biosciences). A stop solution (H_2_SO_4_) was added, and absorbance at 450 nm was measured with reduction at 630 nm using an ELISA plate reader. Results are expressed in optical density. IgM, IgG, IgA and IgE were measured in the serum as previously described.^37^

### 4.5 Luminex

Rat serum concentrations of TGFβ1, TGFβ2, TGFβ3, Eotaxin, MIP‐2, MIP‐1α, IL‐13, IL‐1β, IL‐1α, IL‐2, IL‐4, IL‐5, IL‐6, IL‐10, IP‐10, IL‐12p70, IL‐17A, G‐CSF, GM‐CSF, TNFα, IFNγ, GROα, MCP‐1, MCP‐3 and RANTES were quantified using a multiplex kit (MILLIPLEX MAP; Merck, Burlington, MA and ProcartaPlex; Thermo Fisher, Waltham, MA) according to manufacturer's instructions.

### 4.6 Luciferase immunoprecipitation systems (LIPS)

Luciferase immunoprecipitation systems was used for the detection of auto‐antibodies against cytokines as previously described.^41^, ^42^

### 4.7 qPCR

Total RNA was isolated from cells using a TRIzol reagent (Invitrogen) or an RNeasy Mini Kit (QIAGEN, Hilden, Germany). Total RNA was isolated from organs (spleen, liver, colon and brain) by crushing with Ultra‐Turrax (IKA, Staufen, Germany) using a TRIzol reagent (Invitrogen, Carlsbad, CA), and RNA was reverse transcribed with random primers and M‐MLV reverse transcriptase (Life Technologies, Carlsbad, CA) according to manufacturer's instructions. For rat *Il34* qPCR, TaqMan (Thermo Fisher, Waltham, MA) probes were used (Rn01432380_m1; Thermo Fisher, Waltham, MA). Rat *Gapdh, Il17a, Il1b, Tnfa, Il6, Il22, Ifng, Tgfb, Il10* and *Il12p40* qPCR were performed using the Fast SYBR Green Master Mix (Applied Biosystems, Thermo Fisher Scientific). Probes sequence and associated Tm are summarized in Table S2. The reaction was performed on the Applied Biosystems StepOne system (Life Technologies, Carlsbad, CA). Thermal conditions were as follows: 3 s at 95°C, 30 s at 60°C and 15 s at –5°C of the melting temperature with a final melting curve stage. Calculations were made by the ΔΔ*CT* method.

### 4.8 Histology and immunofluorescence

Rat brains were fixed for 24 h using PFA 4% and conserved by increasing sucrose concentrations and frozen. Sections were performed from paraffin‐embedded tissues and frozen brain tissues. For histology analysis, slides were stained with haematoxylin eosin saffron and analysed by an automated tissue slide scanner (Hamamatsu NanoZoomer Digital Pathology system, Japan) and by confocal microscopy (Nikon A1 RSi, Tokyo, Japan). For brain immunofluorescence, slides were incubated with a purified anti‐CD11b/c (a list of the clones and suppliers of all mAbs used in the study in Table S1) and the staining was revealed using a goat anti‐mouse IgG (H+L)‐AF488 (Invitrogen, Carlsbad, CA). After staining, slides were mounted with a Prolong Gold Antifade Reagent with DAPI (Invitrogen, Carlsbad, CA) before analysis with confocal microscopy. The percentage of positive staining was analysed using ImageJ software.

### 4.9 Cell isolation

Rat spleen was digested by collagenase D (Roche) for 30 min at 37°C; the reaction was stopped by adding .01‐mM EDTA. Mice spleen and rats thymic cells and lymph nodes were isolated by crushing with PBS. Red blood cells were lysed using a lysis solution (8.29‐g NH4Cl, 1‐g KHCO_3_, 37.2‐mg EDTA/1‐L deionized water [pH 7.2–7.4]). Rat TCRαβ^−^SIRPα^+^, TCRαβ^+^CD4^+^CD25^–^, TCRαβ^+^CD4^+^CD45RC^high^, TCRαβ^+^CD4^−^CD45RC^high^, TCRαβ^+^CD4^+^CD25^+^CD127^low^ and TCRαβ^+^CD4^−^CD45RC^low/−^ cells were sorted using an FACS ARIA II (BD Biosciences, Mountain View, CA). Monoclonal antibodies are listed in Table S1.

### 4.10 Phenotypic analysis

Cellular phenotype was analysed on spleen, thymus and blood using the antibodies listed in Table S1. Cells were first gated on their morphology, exclusion of singlets and dead cells by staining with fixable viability dye, eF450 or eF506 (Thermo Fisher, Waltham, MA), then cells were gated on the expression of CD45. Rat subsets were identified as follows: DN (CD4^−^CD8^−^), DN1 (CD4^−^CD8^−^CD25^−^CD44^+^), DN2 (CD4^−^CD8^−^CD25^+^CD44^+^), DN3 (CD4^−^CD8^−^CD25^+^CD44^−^), DN4 (CD4^−^CD8^−^CD25^−^CD44^−^), SP CD4^+^ (CD4^+^CD8^−^), SP CD8^+^ (CD4^−^CD8^+^), IgM and IgD expression were analysed among CD45R^+^CD45RA^+^ cells, pDC (TCRαβ^−^CD4^+^CD45R^+^), cDC (TCRαβ^−^CD4^+/−^CD103^+^), NK (SIRPa^−^TCRαβ^−^CD161^++^), NKT (SIRPa^−^TCRαβ^+^CD161^+^), granulocyte (TCRαβ^−^HIS48^+^RP‐1^+^), macrophage/monocyte (CD68^+^), M1 (CD68^+^CD163^−^) and M2 (CD68^+^CD163^+^). For stimulation, splenocytes were incubated with PMA (50 ng/ml) and ionomycin (1 μg/ml) for 4 h in the presence of Brefeldin A (10 μg/ml). Cells were permeabilized with a Fix/Perm kit (Thermo Fisher, Waltham, MA). Abs were used to stain cells, and fluorescence was measured with a BD FACSCanto II flow cytometer (BD Biosciences, Mountain View, CA), and FlowJo software was used to analyse data.

### 4.11 Proliferation assay

Sorted rat TCRαβ^+^CD4^+^CD25^–^ and TCRαβ^+^CD4^−^CD45RC^high^ Teffs, TCRαβ^+^CD4^+^CD25^+^CD127^low^ and TCRαβ^+^CD4^−^CD45RC^low/−^ Treg cells from *Il34^+/+^* and *Il34^−/−^* rats were plated in duplicate or triplicate with stimulating coated αCD3 (G4.18 at 1, .5 and .25 μg/ml) and soluble αCD28 (JJ319 at 10 μg/ml) mAbs in 100 or 200‐μl complete RPMI‐1640 medium in round‐ or V‐bottomed 96‐well plates, at 37°C and 5% CO_2_. Proliferation of CFSE‐labelled TCRαβ^+^CD4^+^CD25^–^, TCRαβ^+^CD4^−^CD45RC^high^, TCRαβ^+^CD4^+^CD25^+^CD127^low^, TCRαβ^+^CD4^−^CD45RC^low/−^ T cells were analysed by flow cytometry 2 or 3 days later by gating on TCRαβ^+^CD4^+^ cells among living cells (DAPI^−^).

### 4.12 Acute DSS colitis in rats

DSS (TdB labs, Sweden) was dissolved in drinking water at 5.5% and given to 9‐week‐old male rats for 7 days. Weight loss and a disease activity index (DAI = clinical score) were assessed every day. Score is defined as follows: stool consistency – 0 (normal), 2 (loose stool) and 4 (diarrhoea); and bleeding – 0 (no blood), 2 (visual pellet bleeding) and 4 (gross bleeding, blood around anus). At day 7, animals were sacrificed, and colon length was measured and H&E stained to estimate the inflammation.

### 4.13 TNBS‐induced colitis in mice

The model of colitis was induced by the rectal administration of TNBS (100 mg/kg in .1 ml; Merck, Burlington, MA) in 50% ethanol. Animals were weighed daily and were sacrificed 3 days post‐injection to measure colon length.

### 4.14 Experimental autoimmune encephalomyelitis (EAE)

Eight‐week‐old *Il34^+/+^* or *Il34^LacZ/LacZ^* C57BL/6J female mice^12^ were immunized by a subcutaneous injection of 100 μl of an emulsion containing 200 μg of MOG_p35–55_ (GeneCust, Boynes, France) in complete Freunds adjuvant (Merck, Burlington, MA) supplemented with 400‐μg heat‐killed *Mycobacterium tuberculosis* (Thermo Fischer, Waltham, MA). An amount of 200‐ng *Bordetella pertussis* toxin (Merck, Burlington, MA) in 100 μl of PBS was injected intraperitoneally on the same day and 2 days after immunization. Adenovirus, coding for murine IL‐34 or null, were injected i.v. at 4.10^9^IP/100 μL/mice 2 days before the immunization. Rapamycin was i.p. injected every day for 15 days at .25 mg/kg (Rapamune, Pfizer). Animals were monitored daily from day 5 and scored on a 5‐point scale as follows: 0, no symptoms; .5, tip of tail is limp; 1, loss of total tail tonus; 1.5, loss of total tail tonus and hind leg inhibition; 2, loss of total tail tonus and weakness of hind legs; 2.5, loss of total tail tonus and dragging of hind legs; 3, loss of total tail tonus complete and paralysis of hind legs; 3.5, loss of total tail tonus and paralyzed hind legs are together on one side of the body; 4, loss of total tail tonus, hind leg and partial front leg paralysis; 4.5, loss of total tail tonus, hind leg and front leg paralysis, 5: mouse is found dead due to paralysis. Due to ethical considerations mice were sacrificed when they reached grade 4 for 24 h.

### 4.15 Wasting disease

*Il2rg^−/−^* SPD rats aged of 6 weeks were injected via the tail vein with 2.5 × 10^6^ sorted TCRαβ^+^CD4^+^CD45RC^high^ Teff cells from *Il34^+/+^* rats in association or not with sorted TCRαβ^+^CD4^+^CD25^+^CD127^low^ or TCRαβ^+^CD4^−^CD45RC^low/−^ Tregs from *Il34^+/+^* or *Il34^−/−^* rats at a Teffs:Tregs ratio of 3.5:1 and 2.1:1, respectively. A control with a PBS injection was also performed.

### 4.16 3′ Differential gene expression (DGE) RNA‐sequencing

Total mRNA from sorted TCRαβ^+^CD4^+^CD25^–^ and TCRαβ^+^CD4^−^CD45RC^high^ Teffs, TCRαβ^+^CD4^+^CD25^+^CD127^low^ Tregs, TCRαβ^+^CD4^−^CD45RC^low/−^ T cells from *Il34^+/+^* and *Il34^−/−^* rats were extracted using an RNeasy Mini Kit (QIAGEN, Hilden, Germany) and protocol of 3′ DGE RNA sequencing was performed as previously described.^16^ The differential expression *p* values were processed with DESeq2.^43^ The data are available at PRJEB52489.

### 4.17 Immune humanized mouse models

For xenogeneic graft‐versus‐host‐disease (GVHD) model, 1.5 × 10^7^ fresh human PBMC were intravenously injected in 1.5‐Gy‐irradiated NSG mice as previously described.^10^ Human PBMC engraftment was monitored in blood, and GVHD development was characterized by ≥20% body weight loss. For the skin rejection model, human skins were obtained from healthy donors from abdominoplasty surgery, and transplantation was performed as previously described.^10^ A total of 5.0 × 10^6^ PBMCs, allogeneic to the graft, were i.v. injected. A graft rejection score was established from 0 to 5 based on macroscopic observations: 1, the skin starts to peel off; 2, thick skin; 3, scab; 4, edges start to take off; 5, the skin is entirely gone. Osmotic pumps (Alzet, model 1004, Cupertino, CA) were filled with rhIL‐34 (.42 μg/h, i.e. .4 m/kg/d; Thermo Fisher, Waltham, MA or Preprotech, Neuilly‐Sur‐Seine, France) and placed i.p. on the day before the injection of the PBMC. Rapamycin (.4 mg/kg/d for 10 days, Rapamune, Pfizer) was injected intraperitoneally.

### 4.18 Study approvals

All animal care procedures were approved by the Animal Experimentation Ethics Committee of the Pays de la Loire region, France, in accordance with the guidelines from the French National Research Council for the Care and Use of Laboratory Animals (permits numbers CEEA‐PdL‐no. 6, APAFIS #12377, #20640, #2162, #0692, #18724 and #27925. For the DIVAT cohort, all recruited patients gave signed informed consent.

### 4.19 DIVAT cohort of kidney transplanted patients

Serum samples from kidney +/− pancreas transplanted patients were obtained thanks to the ‘Données Informatisées et VAlidées en Transplantation’ DIVAT Biocollection (www.divat.fr, French Research Ministry: RC12_0452, last agreement No 13 334, No CNIL for the cohort: 891735) collected between 2004 and 2012, aliquoted and stored at −80°C. All recruited patients gave signed informed consent. Table S3 recapitulates the clinical characteristics.

### 4.20 Statistics

Mann–Whitney *U* test was used for qPCR, FACS, positive area in immunohistofluorescence and ELISA analysis. Mantel Cox Log Rank test was used to analyse survival curves. Two‐way ANOVA and a Bonferroni post‐test were used to compare weight loss and clinical score between groups. Adapted controls were performed together with the test conditions. Animal numbers were determined with ethical committee agreement.

## CONFLICT OF INTEREST

C.G., I.A. and S.B. have patents registered on IL‐34.

## Supporting information

Supporting Information

## Data Availability

The data that support the findings of this study are available from the corresponding authors upon reasonable request.
