# Supplementary material for: IL‐34 deficiency impairs FOXP3+ Treg function in a model of autoimmune colitis and decreases immune tolerance homeostasis
Source: Clin Transl Med. 2022 Aug 28;12(8):e988. doi: 10.1002/ctm2.988 (PMC9420423; doi:10.1002/ctm2.988)
Supplement: Supplementary file 1 — Supporting Information [file CTM2-12-e988-s001.docx]

**Supplemental Figure 1. Generation of *Il34^-/-^* rats and characterization**. **(A)** Amplicons of *Il34*^+/+^ and *Il34*^-/-^ rats were sequenced showing a C insertion in mutated animals, leading to a shift in the open reading frame and a premature STOP codon (not shown in the electrophorogram). **(B)** Photographs represent the general appearance of the rats at 6 months old (scale bar, 10 cm). **(C)** Littermate *Il34*^+/+^ rats (males, n=3-4 and females, n=3-5) and *Il34*^-/-^ rats (males, n=3-9 and females, n=3-7) were weighed for 100 days. Results are expressed as mean ± SEM. **(D)** Representative photographs show the bone aspect, teeth formation and spleen length (Scale bar, 1 cm). **(E)** Femurs of 8 weeks old males (n=3) and females (n=4) *Il34*^+/+^ or *Il34^-/-^* were fixed in 4% formol and then analyzed by µCT for bone mineral density (BMD) and tissue mineral density (TMD). **(F)** Representative photographs show the morphological aspect of *Il34*^+/+^ (top) and *Il34*^-/-^ (bottom) brains. White arrows indicate the zoom in of the hippocampus and cerebellum showed in Figure 1D. Mann Whitney *U* test or two-way ANOVA and a Bonferroni posttest, ns *p*>0.05.

**Supplemental Figure 2. Quantification of cytokines, chemokines, growth factors, antibodies and histological analysis of *Il34^+/+^* vs *Il34^-/-^* rats (A)** Plasma levels of TGFβ1/2, IL-1α/β, -2, -4, -5, -6, -10, -12p70, -13, -17A, G-CSF, GM-CSF, TNFα, IFNγ, GROα, MCP-1, -3, MIP-1α, Rantes and IP-10 were quantified in 4 months old *Il34^+/+^* (n=9) and *Il34^-/-^* animals (n=14). GM-CSF and IL-6 were not detectable. **(B)** IgM, IgG, IgE and IgA were quantified in the plasma of *Il34^+/+^* (n=7) and *Il34^-/-^* rats (n=7). **(C)** Anti-double strain DNA antibodies were measured at different dilutions (1/2, 1/20, 1/200 and 1/2000) by ELISA (n=6 per group). Results are represented as mean ± SEM. **(D)** Liver, colon, pancreas, kidney and skin sections of *Il34^+/+^* and *Il34^−/−^* rats at 6 months old were stained with HES. Original magnification × 20, scale bar 100 μm. Mann Whitney *U* test or two-way ANOVA and a Bonferroni posttest, ns *p*>0.05.

**Supplemental Figure 3. Immune cell populations are not affected by IL-34-deficiency at resting state. (A)** Absolute numbers of thymic cell subsets were analyzed in 8 weeks old *Il34*^+/+^ (n=7) and *Il34^-/-^* (n=8) rats using markers described in *Supplemental Methods.* Absolute numbers of B, DC, NK, NKT, granulocyte and monocyte/macrophage cell subsets were analyzed in spleen **(B)** and blood **(C)** of >10 months olds *Il34*^+/+^ (n=4-9) and *Il34^-/-^* (n=4-10) rats. **(D)** TCRαβ^+^CD4^+^FoxP3^+^ (CD4^+^ Tregs) and TCRαβ^+^CD4^-^FoxP3^+^ (CD8^+^ Tregs) T cells and **(E)** TCRαβ^+^CD4^+^FoxP3^-^ (CD4^+^ Teffs) and TCRαβ^+^CD4^-^FoxP3^-^ (CD8^+^ Teff)s from total splenic cells from *Il34^+/+^* (n=4-9) and *Il34^-/-^* (n=4-10) rats were analyzed for expression of several markers before and after 3 days stimulation with anti-CD3 (1 ug/mL) and anti-CD28 (10 ug/mL) mAbs. **(F)** Regulatory TCRαβ^+^CD4^+^CD25^+^CD127^low^ (CD4^+^ Tregs) and TCRαβ^+^CD4^-^CD45RC^low/-^ (CD8^+^ Tregs) T cells from *Il34*^+/+^ (n=5-9) or *Il34^-/-^* (n=4-7) rats were sorted and tested for proliferation capacity in response to increasing concentration of anti-CD3 (0.25-0.5-1 ug/mL) and anti-CD28 (10 ug/mL) mAbs after 2 or 3 days of culture. Results are represented as mean ± SEM.

**Supplemental Figure 4. Colitis and EAE autoimmune models in deficient rodents.** (**A**) 5.5% of DSS in drinking water was given for 7 days and colons from *Il34^+/+^,* *Il34^-/-^* or control *Il34^+/+^* rats were harvested to isolate mRNA and RT-qPCR were performed. (**B**) *Il34^+/+^*, *Il34^-/-^* and *Il34^+/+^* control mice weight was followed for 3 days after the intrarectal administration of TNBS (100 mg/kg) in 50% ethanol or ethanol control alone. (**C**) Schematic showing the MOG-induced EAE model in mice. After immunization with the peptide MOG_35-55_ and mycobacterium, a daily clinical score was assessed. Results are represented as mean ± SEM. Mann Whitney *U* test or two-way ANOVA and a Bonferroni posttest, * *p*<0.05; ** *p*<0.01; *** *p*<0.001.

**Supplemental Figure 5. Human cell engraftment analysis in immune humanized mice**. At day 15 post-PBMC injection, FACS analysis was performed on human cells (hCD45) in the blood reflecting the engraftment (n=4-13) in the NSG GVHD **(A)** and skin graft rejection models **(B)**. FACS analysis of human engrafted cells (% of cells) was performed on the blood of IL-34 treated mice (IL-34 pump) vs control mice (PBS-pump), at day 15 post-PBMC injection for the GvHD model (**C**), and at day 30 for the skin graft model (**D**). The percentage of hCD45^+^ cells and of the sub-populations CD3^+^, CD14^+^, CD19^+^ and CD56^+^ cells reflect the engraftment (*right panel*), and a focus on the regulatory CD4^+^ and CD8^+^ T cell compartment is analysed for these two models (n=3-7) (*left panel*). Mann Whitney *U* test, ns *p*>0.05, * *p*<0.05.

**D**

**Supplemental Figure 6. Correlation analysis of kidney transplant survival and IL-34/CSF-1 levels**. **(A)** The percentage of samples positive for IL-34 detectable levels was analyzed among the kidney transplanted cohort and healthy volunteers (HV). Graft survival of patients with an acute rejection episode occurrence according to IL-34 detectability post-Tx (undetectable: < 37.5 vs detectable: >37.5 pg/mL) **(B)** or CSF-1 mean expression (below vs above 1075.83 pg/mL) **(C)**. Log- rank tests, ns *p*>0.05. (**D**) IL-34 was quantified in the serum of healthy volunteers (n=30) and in patients before (Pre-Tx) and after (Post-Tx) transplantation with a stable graft function or having had ≥ 1 episode of acute rejection within the 18 months following Tx. Paired graphs, were generated across all samples analyzed for IL-34 serum levels. 16 and 34 samples were matched Pre-Tx/Post-Tx for patients that will present one or more rejection episode within 18 months or stable within 18 months, respectively.

**Supplemental Table 1.** Antibodies used in this study

| **Marker** | **Clone** | **Provider** |
| --- | --- | --- |
| hCD45 | HI30 | BD Biosciences |
| rTCRαβ | R7/3 | BD Biosciences |
| rCD4 | OX35 | BD Biosciences |
| rCD45RC | OX22 | Hybridoma from ECACC |
| rCD25 | OX39 | In-house |
| rCD127 | 717519 | Biotechne |
| rCD45RA | OX33 | BD Biosciences |
| rCD45R | His24 | BD Biosciences |
| rCD45 | OX1 + OX30 | BD Biosciences |
| rSIRPα | OX41 | In-house |
| rCD161 | 3.2.3 | In-house |
| rCD11b/c | OX42 | In-house |
| rCD103 | OX62 | In-house |
| Granulocytes | RP-1 | BD Biosciences |
| Granulocytes | HIS48 | BD Biosciences |
| rIgM | MARM-4 | In-house |
| rIgD | MARD-3 | In-house |
| rCD8 | OX8 | In-house |
| rCD44 | OX49 | BD Biosciences |
| rCD163 | ED2 | Bio-Rad |
| rMHC-II | OX6 | In-house |
| rFoxP3 | FJK16S | eBiosciences |
| rCD68 | ED1 | Bio-Rad |
| rCD71 | OX26 | In-house |
| rCD27 | LG.3A10 | In-house |
| rCD28 | JJ319 | In-house |
| rCD40L | AH.F5 | Biogen |
| rCD62L | OX85 | In-house |
| rICOS | JTT.1 | In-house |
| rCD122 | L316 | In-house |
| rIL-10 | A5-4 | BD Biosciences |
| rIFNγ | DB-1 | BD Biosciences |
| rCD3 | G4.18 | BD Biosciences |
| mCD3 | 500A2 | BD Biosciences |
| mCD4 | RM4-5 | BD Biosciences |
| mCD45RC | DNL1.9 | BD Biosciences |
| mFoxP3 | FJK16S | eBiosciences |
| mIL-34 | Polyclonal | Biotechne |

**Supplemental Table 2.** Probes used in this study.

| Gene | Primer | Sequence | Tm |
| --- | --- | --- | --- |
| *Gapdh* | Forward  Reverse | AGACAGCCGCATCTTCTTGT  CTTGCCGTGGGTAGAGTCAT | 76 |
| *Il17a* | Forward  Reverse | CTTCTGTGATCTGGGAGGCA  GTCTGTTTAGGACGCATGGC | 78 |
| *Il1b* | Forward  Reverse | GGGATGATGACGACCTGCTA  TGTCGTTGCTTGTCTCTCCT | 75 |
| *Tnfa* | Forward  Reverse | CTTCTCATTCCTGCTCGTGG  GCTACGGGCTTGTCACTCG | 76 |
| *Il6* | Forward  Reverse | GCAAGAGACTTCCAGCCAGTT  CATCATCGCTGTTCATACAATCA | 74 |
| *Il22* | Forward  Reverse | TCTGCCCATCAACTCCCAAT  TTGGCTTTGACTCCTCGGAA | 75 |
| *Ifng* | Forward  Reverse | AGTGTCATCGAATCGCACCTG  TTCTGGTGACAGCTGGTGAAT | 74 |
| *Tgfb* | Forward  Reverse | CTCAACACCTGCACAGCTCC  ACGATCATGTTGGACAACTGCT | 79.5 |
| *Il10* | Forward  Reverse | TGCTATGTTGCCTGCTCTTACTG  TCAAATGCTCCTTGATTTCTGG | 76 |
| *Il12p40* | Forward  Reverse | ATCATCAAACCGGACCCACC  CAGGAGTCAGGGTACTCCCA | 76 |

**Supplemental Table 3.** Description of groups and demographic characteristics of the cohort analyzed for IL-34 and CSF-1 serum levels.

| **IL-34** | | | | | |
| --- | --- | --- | --- | --- | --- |
|  | **HV** | **Rejection patients** | | **Stable patients** | |
|  |  | **Pre-transplantation** | **Post-transplantation** | **Pre-transplantation** | **Post-transplantation** |
| *n* | 30 | 71 | 42 | 101 | 71 |
| *Age (y)* | 43.1±15.8 | 46.3±13.7 | 52±14.3 | 48.7±12.9 | 46.8±12.5 |
| *% of women* | 30% | 46.7% | 26.8% | 35.3% | 32.4% |
| *IL-34 Positive samples (%)* | 13.7% | 18.6% | 22% | 9.9% | 8.5% |
| *CNI* | / | / | 42/42 | / | 24/24 |
| *Tacrolimus* | / | / | 41/42 | / | 23/24 |
| **CSF-1** | | | | | |
|  | **HV** | **Rejection patients** | | **Stable patients** | |
|  |  | **Pre-transplantation** | **Post-transplantation** | **Pre-transplantation** | **Post-transplantation** |
| *n* | 20 | 65 | 22 | 14 | 15 |
| *Age (y)* | 40.7±16.9 | 46.7±13.3 | 53.91±13.1 | 45±11.5 | 42.6±13.1 |
| *% of women* | 29.4% | 49.2% | 39.1% | 28.6% | 33.3% |
| *CNI* | / | / | 16/16 | / | 9/9 |
| *Tacrolimus* | / | / | 16/16 | / | 8/9 |
